# Moment Fitting for Parameter Inference in Repeatedly and Partially Observed Stochastic Biological Models

**DOI:** 10.1371/journal.pone.0043001

**Published:** 2012-08-10

**Authors:** Philipp Kügler

**Affiliations:** Mathematical Methods in Molecular and Systems Biology, Johann Radon Institute for Computational and Applied Mathematics, Linz, Austria; Université de Nantes, France

## Abstract

The inference of reaction rate parameters in biochemical network models from time series concentration data is a central task in computational systems biology. Under the assumption of well mixed conditions the network dynamics are typically described by the chemical master equation, the Fokker Planck equation, the linear noise approximation or the macroscopic rate equation. The inverse problem of estimating the parameters of the underlying network model can be approached in deterministic and stochastic ways, and available methods often compare individual or mean concentration traces obtained from experiments with theoretical model predictions when maximizing likelihoods, minimizing regularized least squares functionals, approximating posterior distributions or sequentially processing the data. In this article we assume that the biological reaction network can be observed at least partially and repeatedly over time such that sample moments of species molecule numbers for various time points can be calculated from the data. Based on the chemical master equation we furthermore derive closed systems of parameter dependent nonlinear ordinary differential equations that predict the time evolution of the statistical moments. For inferring the reaction rate parameters we suggest to not only compare the sample mean with the theoretical mean prediction but also to take the residual of higher order moments explicitly into account. Cost functions that involve residuals of higher order moments may form landscapes in the parameter space that have more pronounced curvatures at the minimizer and hence may weaken or even overcome parameter sloppiness and uncertainty. As a consequence both deterministic and stochastic parameter inference algorithms may be improved with respect to accuracy and efficiency. We demonstrate the potential of moment fitting for parameter inference by means of illustrative stochastic biological models from the literature and address topics for future research.

## Introduction

The traditional approach to modelling of biological reaction networks is based on deterministic mass action kinetics in which the time course of the species concentrations averaged over the population is described by a set of coupled ordinary differential equations [1], often referred to as the macroscopic rate equations. For the description of intra-cellular processes characterized by a low number of reacting molecules the stochastic modelling approach [2] is an alternative that explicitly takes the discreteness and stochasticity of chemical kinetics into account. In well-mixed conditions the system dynamics are captured by the Kolmogorov differential equation, also referred to as the chemical master equation, for the transition probability kernel of a continuous time Markov process with discrete state space. Numerical solutions of the master equation, even after projection to finite state space [3], are computationally expensive, but realizations of the stochastic process can be achieved by the Gillespie algorithm and its variants [4], [2]. A stochastic differential equation approximation to the true process is given by the chemical Langevin equation [5], the associated Fokker Planck equation then describes the probability density function of the continuous state variable. An alternative approximative description is the linear noise approximation [6] that features a partial differential equation for the probability distribution of the fluctuations around the deterministic part governed by the macroscopic rate equation.

Parameter estimation in differential equation models is a classic nonlinear inverse problem that arises in a variety of scientific, industrial and financial applications and is approached both in deterministic and statistical ways [7], [8], [9], [10]. The advances of experimental biology even at the single cell level [11], [12] along with the ever growing quality and amount of species concentration data have also stimulated recent interest in the inference of reaction rate parameters in kinetic biological models. A common feature of many parameter estimation methods is the comparison of time series data with parameter-dependent model predictions. For instance, [13], [14], [15] compare time series data with the solution of the macroscopic rate equation in the minimization of unregularized and regularized least squares functionals by deterministic and stochastic optimization routines, [16] compares finite differences of time series data with the drift term of the chemical Langevin equation in Bayesian inference, [17] compares time series data with the solution of the macroscopic rate equation or with averaged outcomes of the Gillespie algorithm in approximate Bayesian inference, [18] compares time series data with the solution of the macroscopic rate equation in maximum likelihood estimation, [19] compares time series data with the mean component of the linear noise approximation in Bayesian inference, [20] compares probability density or cumulative density functions obtained from the data with their counterparts constructed from repeated realizations of the Gillespie algorithm, and [21], [22], [23] sequentially compare time series data with the solution of the macroscopic rate equation when applying extended Kalman filters or nonlinear observers. For inferring the rate parameters 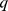 from a time series data vector 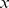, some of the available approaches explicitly take parameter dependent predictions of the mean 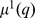 (the first moment) as well as of higher moments, e.g., of the variance 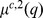 (the second central moment), of the state variable 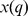 into account, for instance, for building the (simplified) likelihood function
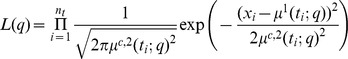



for a multivariate normal distribution or a weighted sum of squared residuals
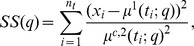



see [24], [19], [16], [25]. However, an adjustment of model parameters in order to actually also *fit higher sample moments* derived from time series data, e.g., by computing the distance between the sample variance 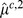 and the variance model prediction 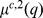, so far has - to the best of our knowledge - not been considered. Furthermore, studies of parameter sensitivities and identifiability [26], [27], [28], [29], [30] typically are based on macroscopic rate equation models where the Hessian matrix of 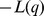 or 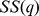 (usually with 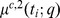 replaced by parameter independent weights) to be analyzed involves only a comparison of the data 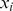 with the first order moment 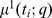. Small or zero eigenvalues of the Hessian at a minimizer point to small or even vanishing curvatures of the landscape function in parameter space, a situation called parameter sloppiness [27] as parameters then are only poorly constrained by the data and not uniquely identifiable. Parameter sensitivities have also been studied for stochastic chemical kinetics models [31] based on the linear noise approximation, still the distance between sample and theoretical moments is not part of this analysis. Finally, the design of experimental stimulation protocols for improving the parameter identifiability in macroscopic equation models has been studied in [32].

In this paper we present a moment fitting approach to parameter inference in stochastic biological models from time series data that to the best of our knowledge has not been studied before. First, we suppose that the state variable vector of molecule numbers can be partially - both with respect to time and state variable components - and repeatedly, say 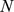 times, observed such that sample moments 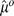 up to some order 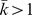 of interest for the observable components can be computed from the data. Experimental techniques that may provide molecule number information are presented in [33], [34], [35], [36]. Second, we consider closed systems of ordinary differential equations
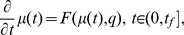


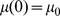



that describe the time evolution of parameter dependent moment approximations 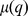 up to the order 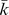 and can be obtained from the chemical master equation via moment closure techniques, the Fokker Planck equation or the linear noise approximation [37], [38], [39], [40], [41], [42]. Now, let 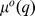 denote those components of 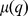 that depend only on the observable state variables. For solving the parameter inference problem we then suggest to utilize the distance 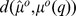 between the sample moments 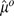 and the equation output 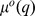 in global and local minimization techniques or approximate Bayesian methods. For 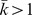 the span of the eigenvalues of the Hessian matrix of 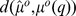 may be strongly reduced in comparison to cost functions that only involve the first moment (the mean) such that problems of non-identifiability or parameter sloppiness can be relieved or even overcome. That way the efficiency and accuracy of distance based parameter inference strategies may be enhanced by higher order moment fitting. In comparison to [20], where the focus is on a comparison of probability density functions rather than on a comparison of statistical moments, model predictions based on the above mentioned ODE system are computationally much cheaper than those based on repeated realizations of the Gillespie algorithm. Analytic expressions for the steady state mean and variance of a scalar variable have been used in [35] in order to obtain initial guesses for a parameter inference routine based on comparing experimental histograms from stationary data with model predictions. Still, the latter are obtained as in [20] from repeated simulations of the (stationary) chemical master equation. The moment fitting approach presented in our paper handles time series data and involves only the solution of a single moment ODE system rather than repeated stochastic simulations. This reduces the computational costs and allows one to build parameter sensitivities for gradient based optimization and solution analysis. In a sequence of illustrative examples with simulated data from the literature we demonstrate potential benefits of our approach and address future challenges.

## Results

We studied the concept of moment fitting for parameter inference in stochastic biological models from time series data by means of three reference examples, see Materials and Methods for all model details, and chose 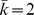 as highest moment order in all of our tests.

### Results for Linear Birth and Death Process

The Kolmogorov differential equation (11) in this example reads as
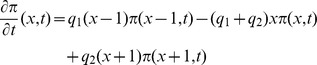



and implies the ODE system
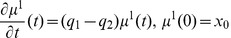
(1)

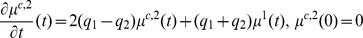



for the mean 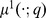 (the first moment) and the variance 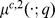 (the second central moment) approximation of the discrete state variable 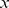, see Supporting Information S1 for a derivation. Due to the linearity of the rate function 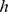 with respect to 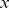, the very same system (1) is obtained if the true stochastic process is approximated by the diffusion approximation (12), (13) or by the linear noise approximation (22). Furthermore, 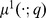 and 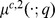 coincide with the true moments 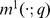 and 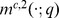. Another consequence of the linearity of 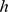 is that (1) admits an analytical solution given by
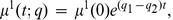
(2)

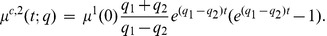
(3)


**Figure 1:**
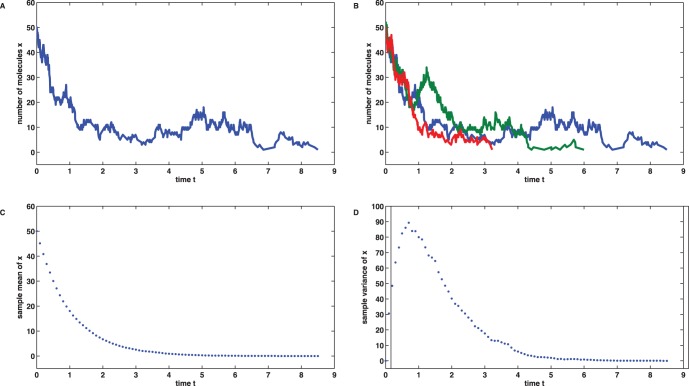
Simulation and data of the linear birth death process. (A) A single realization of the true stochastic process with 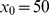 and rate parameters 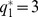, 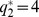. (B) Three (out of 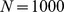) realizations of the true stochastic process. The process is finished as soon as the case 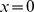 occurs. (C) Sample mean 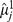 at the discrete observation times 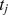. (D) Sample variance 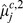 at the discrete observation times 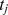.

For the purpose of data generation we have simulated the true stochastic process 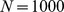 times by means of the Gillespie algorithm [4]. Figure 1 B shows 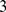 (out of 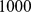) realizations as example, Figures 1 C and D display the sample mean 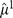 and the sample variance 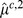 at the process observation times 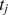 derived from the data.

**Figure 2:**
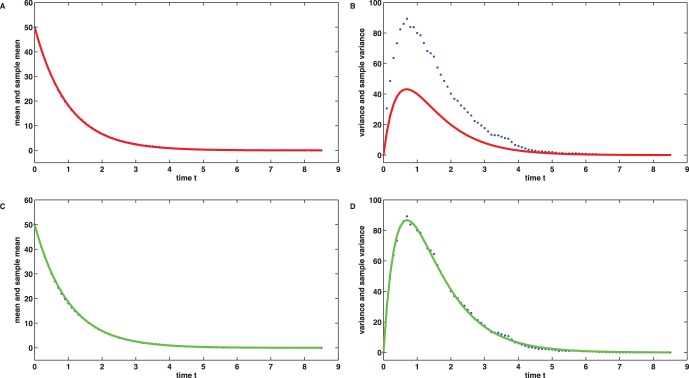
Moment fitting for the linear birth death process. (A) The minimization of the cost function (4) yields a perfect match between the mean data and the first moment expression 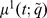, but a parameter result 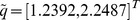 with large deviations from the true values 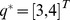. (B) As a consequence, the variance data cannot be explained by the second order moment expression 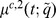. (C) The alternative minimization of (5) once more yields a perfect match between the mean data and 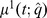 but in addition the significantly improved parameter estimate 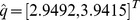. (D) As the fitting of the central second order moment has been explicitly taken into account by (5), now also the variance data is reproduced by 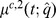.

In order to infer the rate parameters 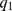 and 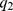 we first utilized the cost (or distance) function
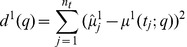
(4)


for a comparison of the sample mean with the analytic mean expression (2). Choosing the initial parameter guess 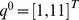 and the MATLAB [42] trust region optimization algorithm with default setting we obtained the parameter solution 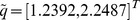 after 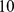 iteration steps. Though the mean concentration data are perfectly fit by 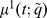, see Figure 2 A, the solution 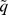 strongly deviates from the true parameter values 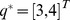. If 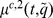 is used to predict the sample variance, large errors can also be observed in the data space, see Figure 2 B.

The problem of non-identifiability is overcome if not only the first moment but also the second central moment is fitted to the available data, e.g., by minimizing the cost function
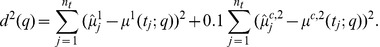
(5)


Again starting from 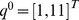 the minimization of 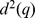 after 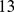 iteration steps led to the parameter estimate 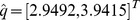 which is nearly identical to the true solution 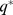. Figures 2 C and D indicate the quality of the data fit by the analytic moment expressions 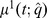 and 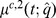.

**Figure 3:**
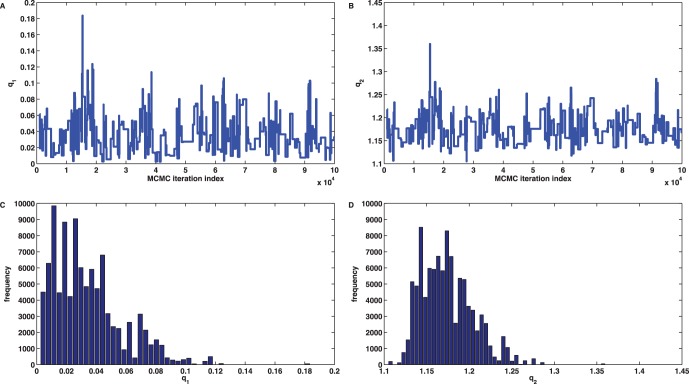
MCMC Metropolis random walk for the linear birth death process. Figures (A)–(F) show the output of a MCMC Metropolis random walk for the inference of the marginal parameter posterior density distributions using the likelihood function (6). The prior parameter distributions were chosen as 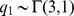 and 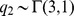, and the candidate parameter vector 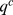 at stage 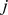 was given by 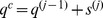 with random innovations 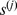 drawn from 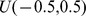. The iteration number of the sampler was set to 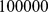 and the first 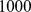 steps were discarded as burn-in and ignored in the monitoring plots (A)-(F). (A,B) Trace plots of the marginal posterior distributions for 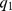 and 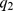 with only small movement around the mean values 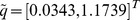, largely deviating from the true values 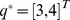. (C,D) Frequency histograms with 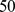 bins corresponding to the trace plots of (A,B).

In a further test, we utilized the likelihood function
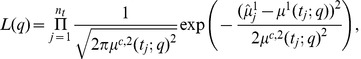
(6)


which compares 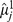 to 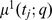 but *does not involve* the error between 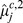 and 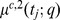, in a MCMC Metropolis random walk algorithm [2]. Even if we chose the favourable gamma distributions
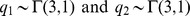



as priors for the parameters, the algorithm failed to yield acceptable marginal posterior density distributions due to the ignorance of the sample variance 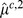, see Figure 3 for details.

### Results for Dimerisation Kinetics

Based on the Fokker Planck equation (13) of the diffusion modelling approach
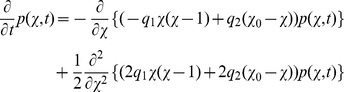



the normal moment closure technique yields the nonlinear ODE system
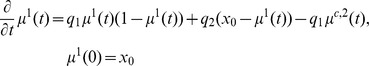


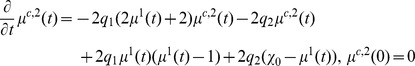



for the (approximative) mean 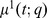 and the (approximative) variance 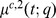 of the continuous state variable 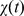, see Supporting Information S1 for the derivation. The true stochastic process was simulated 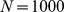 times by means of the Gillespie algorithm [4] with initial molecule number 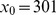 and rate parameters 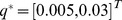.

**Figure 4:**
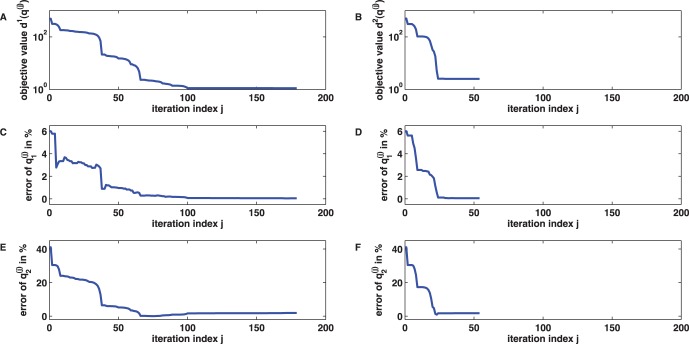
Iterative minimization for inference of the dimerisation process parameters. Iterative minimization of the cost functions (7) and (8) using the MATLAB trust region algorithm with default settings and and initial guess 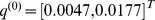. The gradient information both for (7) and (8) was provided by means of the adjoint method in order to avoid error-prone finite differencing. (A) Plot of the value of the cost function (7) at the iterate 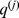. The optimization algorithm terminates after 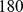 (outer) iteration steps and yields the minimizer 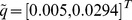. (B) Using the cost function (8) instead of (7), the algorithm already terminates after 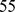 (outer) iteration steps and yields the minimizer 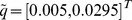. (C,E) Plots of the relative errors 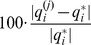, 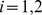 show that convergence to the true parameter vector 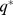 is obtained (up to a negligible error in 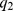) if (7) is chosen as objective function. (D,F) Parameter convergence is also obtained if (8) is chosen instead of (7). However, parameter convergence is much faster in this case.



We first only focused on the sample mean data 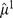 and minimized the least squares objective function
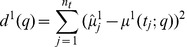
(7)


by means of the MATLAB [42] trust region routine. The initial parameter guess chosen was 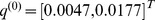, for any 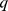 the model predictions 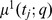, 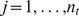, were obtained by solving the above mentioned ODE system, and the gradient of 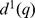 was provided by means of the adjoint method, see Supporting Information S2. Figures 4 A,C,E show that convergence of the iterates towards 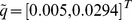 is obtained after 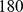 iteration steps. Though the mean data in this example is sufficient to obtain reliable parameter estimates (also for more distant initial guesses), a significant computational speed up is gained if not only the mean data but also the variance data 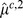 is taken into account. Figures 4 B,D,F display the performance of the same optimization algorithm with identical initial guess if applied to the minimization of the alternative objective function

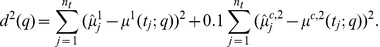
(8)The accuracy of the parameter estimate 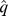 obtained with (8) is the same as of 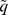 obtained with (7), however, convergence is now already achieved after 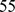 iterations. The outcome 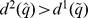 is solely due to the additional variance term in (8) and does not allow for a comparative judgement of the inferred parameters.

### Results for 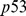 Signalling System

**Figure 5:**
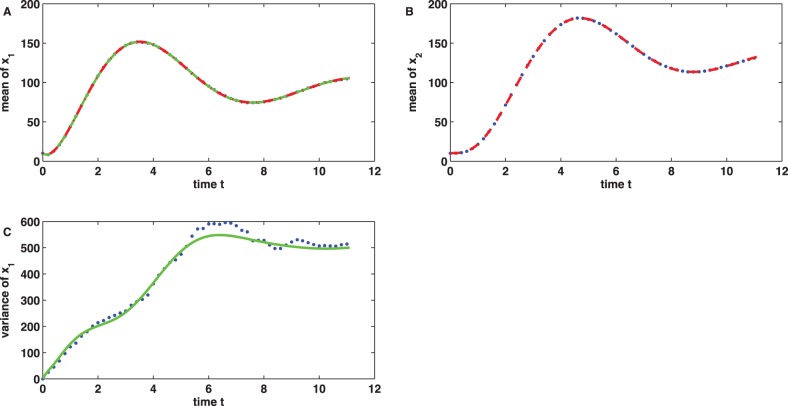
Moment fitting for the 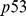 model. (A, B) The minimization of the cost function (9) yields a perfect match between the sample mean data 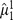, 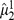 and the outputs 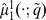, 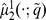 (red curves) of the linear noise approximation for the 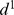-minimizer 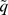. (C) The plot shows the variance data 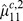 and the model output 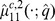 (green curve) calculated with the minimizer 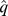 of the cost function (10). Though the approximation errors are more pronounced the consideration of 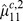 in the inference task yields a improved parameter estimate 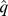.

**Figure 6:**
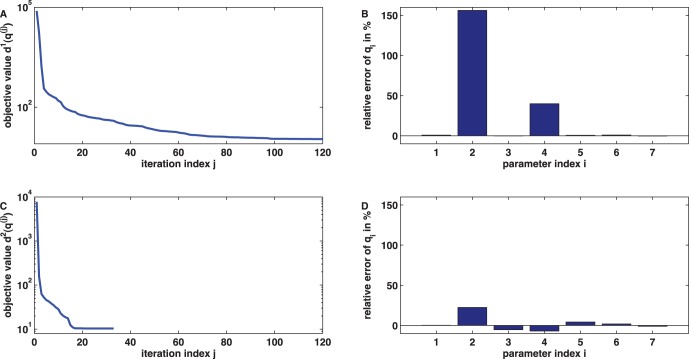
Iterative minimization for inference of the 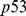 model parameters. Iterative minimization of the cost functions (9) and (10) using the MATLAB trust region algorithm with default settings, the initial guess 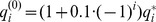, 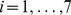, and the adjoint method for providing the gradient information. (A) Plot of the value of the cost function at the iterate 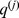. The optimization algorithm terminates after 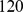 (outer) iteration steps and yields the minimizer 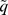. (B) Plot of the relative errors of 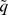 in 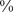 showing a huge deviation from the true parameter 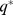 in the second and fourth components. (C) Plot of the value of the cost function  at the iterate 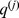. The optimization algorithm terminates after only 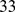 (outer) iteration steps and yields the minimizer 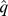. (D) The quality of the parameter estimate 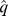 has significantly improved in comparison to 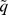.



The linear noise approximation (14) yields a nonlinear ODE system describing the temporal development of the mean approximation 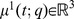 and the covariance matrix approximation 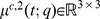. For data generation, the true stochastic process was simulated 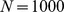 times by means of the Gillespie algorithm [4] with the initial molecule numbers 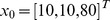 and the rate parameter vector 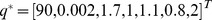. First, we supposed that the two components 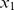 and 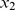 of the state vector 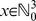 can be observed. The minimization of the objective function
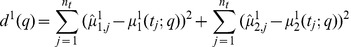
(9)


with the initial guess 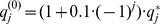, 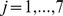, showed that the corresponding sample mean data 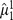 and 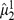 are not sufficient to identify the true vector 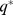. Though the parameter estimate 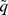 obtained after 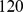 iterations is able to reproduce the data, see Figures 5 A and B, it features a maximal relative error of 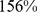 in its second component, see Figures 6 A and B for details. For comparison, we next supposed that only the component 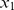 is amenable to observations but build both the corresponding mean estimate 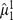 and the variance estimate 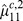 from the data, see Figures 5 A and C. The minimization of the objective function

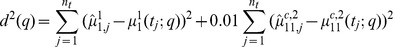
(10)(with the same initial guess and MATLAB trust region algorithm as for (9), gradient information again provided by the adjoint method) led - after only 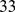 iterations - to an improved parameter estimate 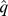 whose maximal relative error (again in the second component) was reduced to 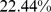, see Figures 6 C and D for details.

## Discussion

**Figure 7:**
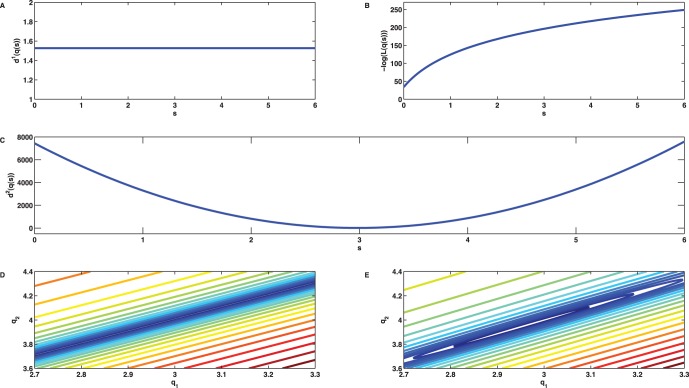
Distance functions for the linear birth death process. The problem of minimizing the least squares error between the sample mean and the analytic mean 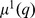 has infinitely many global solutions 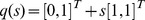 with 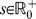. (A) Values of the cost function 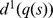 from (4) for 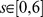. (B) Values of the negative log-likelihood function 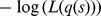 for 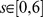 with 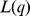 as in (6). The likelihood 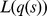 is maximal for 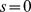 which explains the observation made in Figure 3. (C) Values of the cost function 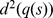 from (5) for 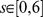. The function also measures the distance between the second order sample and analytic moments and as a consequence admit a unique global minimum at 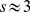 corresponding to the true parameter solution, i.e., 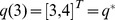. In this example, 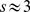 and 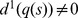 are due to the finite sampling number 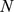. (D) Level sets of the cost function 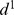 indicate extreme parameter sloppiness and infinitely many parameter solutions. (E) Ellipsoidal level sets of the cost function 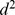 in the neighbourhood of the true solution 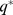 indicate its unique identifiability.



Our numerical tests by means of reference models from the literature show that comparing sample moments with theoretical moments may enhance parameter identifiability and the performance of parameter identification algorithms in comparison to mean-only approaches. These observations can be explained by an examination of the cost functions that have been used for the parameter inference. In the linear birth death example the mean expression (2) shows that any parameter combination 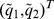 with 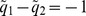 equally well explains the sample mean data as the true parameter vector 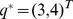. The failure of the MCMC approach based on the likelihood (6) can be understood from plotting the negative log-likelihood along the line 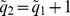 which assumes its minimum at the boundary imposed by parameter positivity and far away from 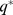. With respect to the cost function (4) we have 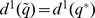, see Figure 7 A. This non-identifiability is also revealed by a parameter sensitivity analysis [26], [27], [28], [29], [30] based on an eigenvector decomposition of the Hessian matrix

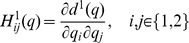



of 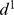. If evaluated at 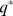 the two (normalized) eigenvectors are 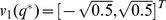 and 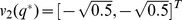 with corresponding eigenvalues 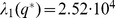 and 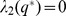. While 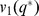 points towards the direction of maximal curvature (or the stiff direction), 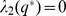 indicates that there is no curvature at all along the direction of 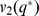 (the soft or sloppy direction). A large eigenvalue spectrum, which in this extreme example spans infinitely many decades, is referred to as parameter sloppiness. The results of the analysis of the Hessian are also reflected in Figure 7 D which plots the level sets of (4). The cost function is minimal on a whole line, whose direction is given by 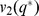, rather than on an isolated point. The situation significantly improves if instead of (4) the cost function (5) is chosen which also takes the mismatch between the sample covariance and the analytic expression (3) into account. The eigenvector decomposition of the Hessian matrix
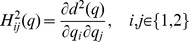



of (5) evaluated at 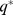 yields the eigenvectors 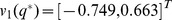 and 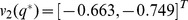 with corresponding eigenvalues 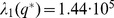 and 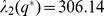. Though the directions of largest and smallest curvature are similar as before, the Hessian now is regular with a narrow span of the eigenvalues. A plot of the level sets, see Figure 7 D, indicates that - as opposed to 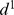 - the function 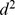 admits a landscape with a sharp trough in the neighborhood of the isolated minimizer. The unique parameter identifiability is also evident from the quadratic behaviour of the cost function 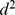 along the line 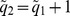 (with direction 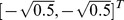), see Figure 7 C, with its global minimizer corresponding to 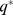. These results hold true if the weighting parameter 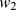 in the definition of 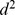 is changed from 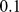 to, e.g., 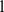 or 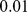.

**Figure 8:**
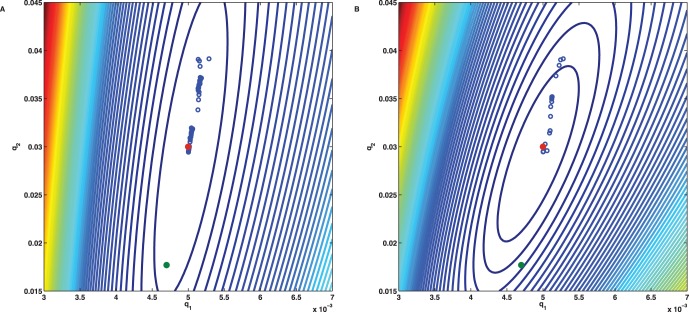
Level sets of the cost functions for the dimerisation process. (A) The level sets of the cost function 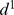 reveal an elongated and flat valley in which the iterates of gradient based optimizers may only slowly converge towards the minimizer 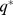. (B) The level sets of the cost function 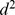 form ellipses with smaller ratio of major axis over minor axis and correspond to a more pronounced trough. As a consequence the iterates converge faster towards 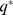.

For the dimerisation example the level sets of the cost function (7) are shown in Figure 8 A. In this example the mean sample data is sufficient to uniquely determine the model parameter vector 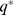, at least if a nearby initial guess is chosen. But even then the iterates of a gradient based optimizer may be forced to slowly wander along the elongated and flat valley of 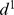 before they reach the unique minimizer. In comparison, the level sets of the alternative cost function (8) show a considerably smaller ratio between the major and minor axes of the ellipses, see Figure 8 B, such that the iterates may faster approach the minimizer. The condition number of the Hessian matrix 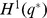 for 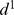 is given by 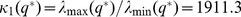, while for the Hessian 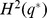 for 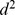 a reduction by 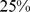 is achieved. The observed algorithmic improvement is in agreement with gradient based optimization theory [43] according to which the rate of convergence improves if the condition number of the Hessian matrix, also reflected in the contour plots of the level sets, decreases.

Similar conclusions can be drawn from the 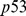 example in which we put focus on the practicability of our approach in case of partial state observations. The parameters in this example have different units and varying scales. As a consequence we build the Hessian matrices of the cost functions 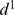 and 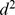


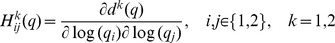



by differentiation with respect to 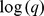 in order to take relative changes in parameter values into account [27]. A relative comparison of the two condition numbers 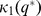 and 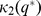 yields
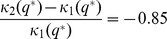



which corresponds to a reduction of the eigenvalue ratio 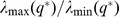 by 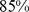 when using 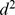 instead of 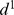. This improvement is even more pronounced if the derivatives in the Hessian are taken with respect to 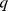 and is also clearly reflected in Figure 6.

Based on accepted mathematical descriptions of stochastic reaction networks in well-mixed conditions we have introduced the concept of moment fitting for parameter inference from time series data of repeated measurements. In numerical tests for simple reference examples we observed that if the common comparison of sample mean data with the parameter-dependent mean expression derived from the model is augmented by consideration of higher moments such as covariance, improvements both with respect to parameter estimate accuracy and algorithmic efficiency are achieved.

Our approach offers opportunities for extension and further research. In case of larger models and real data, then also affected by external noise, measures against data instability and overfitting become necessary. The regularization theory of nonlinear inverse problems [7], [44] suggests the application of stopping rules for the iterative minimization of cost functions in dependence on the quality of the data. Stopping for overfitting avoidance is also recommended from the view point of data mining [35], then also referred to as pruning. Pruning in order to obtain a simple description that still fits the data can also be realized by augmenting the cost function with sparsity enforcing 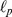-priors or penalties, i.e.,
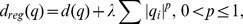



see [45], [46], [47]. In the context of parameter inference for biochemical reaction networks 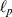-penalization has been previously used in [48], [49], [50]. As the eigenvalues of the Hessian of a cost function 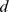 also reflect the degree of ill-conditioning of the parameter inference problem, our examples show that even the pure consideration of higher moments may have a beneficial effect with respect to the amplification of measurement errors. Other possible expansions of the moment fitting approach include the consideration of more elaborate state observation operators, unknown initial conditions or reaction rate parameters that themselves are treated as stochastic quantities.

In our examples we used squared errors to build the distance functions. This not only allowed us to numerically demonstrate the advantages of moment fitting but also to give explanations of the results in terms of Hessian matrices which in the squared error case can be computed in a straightforward manner. Furthermore, the squared error approach enables the straightforward utilization of the adjoint method, see Materials and Methods, for providing gradient information to the optimizer. Still, as moments of different order may be correlated [51], distance functions based on generalized least squares [52] might be an alternative in more demanding situations which then would require to provide covariance estimates for the moments to be fitted. Another idea is to use cost functions that are motivated by 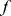-divergences [53] for the comparison of probability distributions such as the Hellinger divergence or the Kullback-Leiber divergence. For instance, the KL-divergence for multivariate normal distributions would suggest the cost function
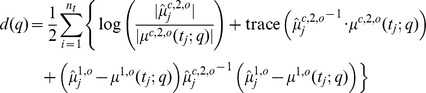



but then poses severe challenges with respect to an efficient computational realization.

The moment fitting approach presented in this paper rests upon the availability of sufficient data for the estimation of higher moments as only then more traditional mean-only approaches may be outperformed. This raises the question of selecting the sample size 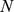 in the design of the experiments. In order to go beyond heuristics it is the goal of our future research to combine error estimates of sample moments, e.g.,
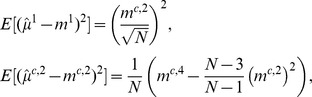



see [54], with error estimates for the theoretical moment approximations described by the ODE system (21), see [55], [56], [41], [37] for preliminary results. An integrative error analysis may then also guide the choice of the moment order 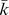 of truncation and the choice of the factors 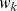 in weighted cost functions.

## Materials and Methods

### Modelling Strategies

In well-mixed conditions a network of 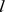 coupled chemical reactions 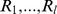 involving 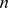 chemical species 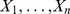 can be characterized by the formalism [2]

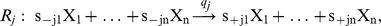



where the integers 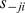 and 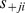, 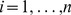 denote the numbers of molecules consumed and produced in a single step of reaction 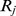. If 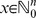 represents the vector of species molecule numbers and 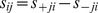 denote the components of the stoichiometric matrix 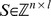, then the state vector is updated according to 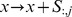 whenever reaction 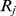 fires. Each reaction 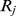 is associated with a rate law (or hazard function) 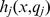 and a stochastic rate constant 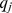.

Let 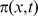 denote the probability of being in state 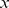 at time 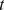 given the initial condition 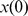. Then, the time evolution of 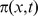 is described by the Kolmogorov differential equation (or chemical Master equation)
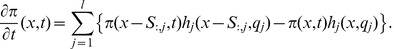
(11)


With 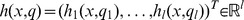 the diffusion approximation to the true process is based on the chemical Langevin equation [5]

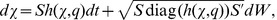
(12)


where 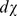 is the change in state 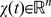 in an infinitely small time interval 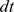 and 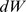 is the increment of a 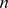-dimensional Wiener process. In the stochastic differential equation (12) the stochastic perturbations are modelled by a state and rate parameter dependent Gaussian noise and the associated probability density function 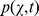 is described by the Fokker-Planck equation
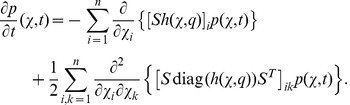
(13)


An alternative approximative description of the stochastic process is given by the linear noise approximation [6] which is derived from a Taylor expansion of (11) in powers of 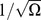 where 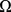 denotes the volume of the reactive system. This leads to a decomposition of the molecule concentration vector 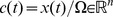 according to
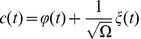
(14)


into a deterministic part 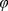 that solves the macroscopic rate equation
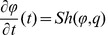
(15)


and a stochastic process 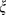 described by a linear diffusion equation
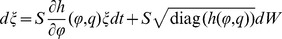



with the increment 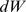 of a 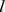-dimensional Wiener process.

Finally, the deterministic modelling approach [1], [57] ignores (if the justifying assumptions are satisfied) random fluctuations due to the stochasticity of the reactions and describes the time course of the species concentration vector 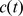 by the set of ordinary differential equations
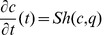
(16)


which corresponds to (15) with the setting 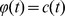.

### Moments of the Random State Variable

Depending on the chosen modelling approach the state variable of a stochastic biochemical reaction network is described as a discrete or a continuous random quantity. In the discrete case associated to the Kolmogorov differential equation (11) the first order moments [58] of the 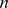-dimensional state variable 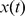 are given by
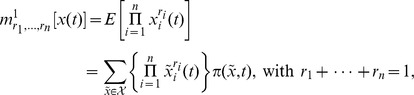



where 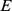 is the expectation operator and 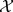 denotes the countable state space. Using this formalism the 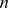-dimensional mean vector 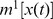 of 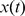 is described by
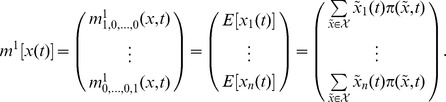



In general, the 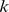-th order moments [58] are given by
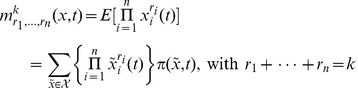
(17)


and the 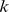-th central moments [58] are given by
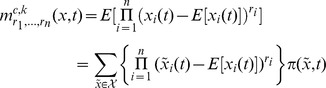



with 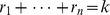. For instance, the covariance matrix 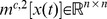 of 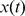 is described by the 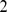-nd order central moments according to
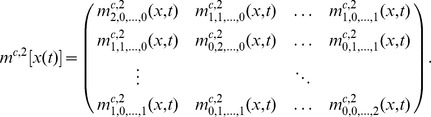



In case the state space of the biological system is modelled as a continuous random variable 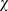 its moments are described the same way after switching from the summation over a probability mass function 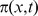 to an integration over a probability density function 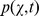 as, e.g., defined by the Fokker Planck equation (13). For instance, the 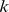-th order moments then are given by
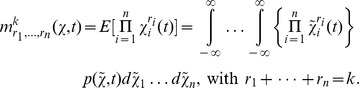
(18)


### Differential Equations for the Moments

Based on the differential equations (11) and (13) for the probability mass and density functions, differential equations that approximatively describe the time evolution of the 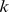-th order moments (17) and (18) or their centered counterparts can be derived. For instance, the time evolution of the mean vector 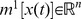 of the discrete state 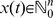 is given by
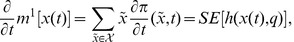
(19)


see [2], [37]. Note that (19) corresponds only to the deterministic rate equation (16) in case of a propensity function 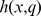 that is linear in 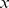 as
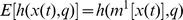



holds only then. Another example is the time evolution of the 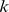-th order moments of a continuous state variable 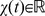 which based on the Fokker Planck equation (13) for 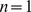 is given by
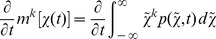


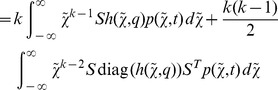


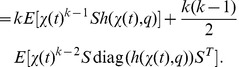
(20)


The simple example 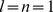 with 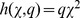 in (20) yields.
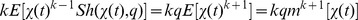



and implies the dependency of the ordinary differential equation for 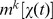 on the higher moment 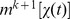. This type of dependency is typical for the moments 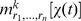 or 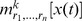 whenever 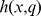 is a nonlinear function of 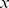 and may render the *exact* differential equations for their time evolution impossible to solve analytically or numerically. The problem may be overcome by the technique of moment closure [37], [38], [39], [40], [41] which sets moments or central moments above a certain order 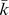 of interest equal to zero or alternatively replaces them by expressions depending only on moments up to order 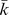. As a result one obtains (manually or supported by symbolic computation tools [39], [59]) a self-contained (or closed) set of coupled ordinary differential equations
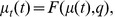
(21)


in which 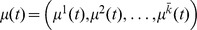 or 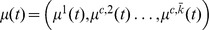 *approximatively* describes the time evolution of the true moments 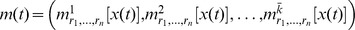 or 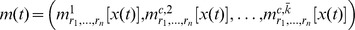. In our notation we emphasize the dependency of 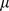 on the rate parameters by writing the solution of (21) (to be supplemented by appropriate initial conditions) as 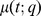. The final form of (21) depends on the underlying modelling approach, the choice of 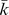 and the choice of the closure technique.

As an alternative to the moment closure approach, a closed form (21) for the approximative description of the moment time course for 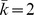 can be obtained from the linear noise approximation [6], [31], [19] which with 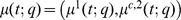 as approximation of 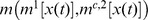 yields the nonlinear ODE system
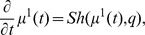


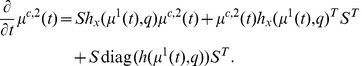
(22)


Here, 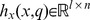 denotes the Jacobian matrix of 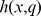 with respect to 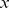.

### Test Models

#### Linear birth and death process

A classic and illustrative reaction system widely studied in the literature is the linear birth and death process [60], [58], [2] for the species 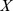 with molecule number 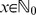. The birth and death reactions are given by
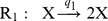


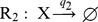



with the associated stoichiometric matrix 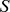 and the rate functions 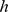


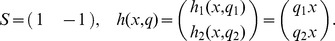



In all simulations of the discrete stochastic dynamics of the model we chose the rate parameters 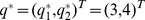 and the initial condition 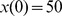.

With respect to the parameter inference problem we suppose that the state variable
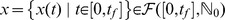



can be only partially observed at the 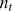 discrete times
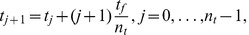
(23)


obtained from an (without loss of generality) equidistant discretization of the time interval 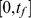 with 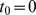. Then, this gives rise to a state observation operator
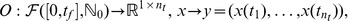
(24)


such that the partial state observation can be compactly described as 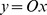. Here, 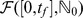 denotes the set of all functions from 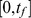 to 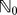. In the example we chose the setting 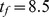 and 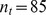. In particular, we do not consider the times and types of the reactions that are fired during the realisation of the stochastic process as amenable to our observations.

#### Dimerisation kinetics

A simple reaction system featuring a nonlinear rate function is the dimerisation process [2], [39]. For the species 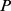 and 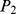 with molecule numbers 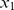 and 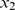 we consider
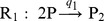


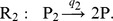



The conservation of the total number 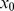 of molecules
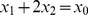



allows to formulate the stoichiometric matrix 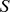 and the rate functions 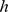 in terms of 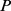 only, i.e., with 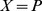 and 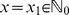 we obtain
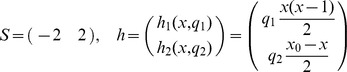



for a one-dimensional state space. In all simulations of the discrete stochastic dynamics of the model we chose the rate parameters 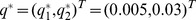, the total molecule number 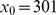 and the initial condition 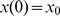. With respect to the parameter inference problem we chose the same observation operator 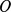 as in (24), now with the setting 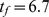 and 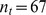.

#### 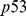 Signalling System

For testing the practicability of our approach in case of partial state observations we have chosen a model for the 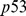 signalling system which features a feedback loop between the tumor suppressor 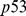, the oncogene 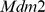 and its precursor 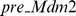. The model was introduced in [61] and also studied in [31]. With 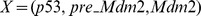 and the associated vector 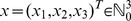 of molecule copy numbers, its stoichiometric matrix 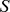 and rate functions 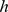 are given by
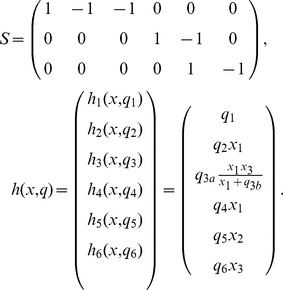



In all simulations of the discrete stochastic dynamics of the model we chose the rate parameters 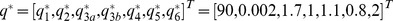 and the initial conditions 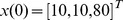. With respect to the parameter inference problem we chose the time discretization as in the previous examples and supposed that either the component 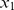 or both the components 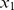 and 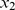 are amenable to observations. The corresponding observation operators are
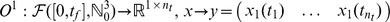



and
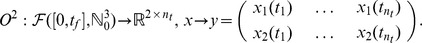



The time observation parameters were chosen as 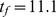 and 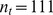.

### Data Generation and Sample Moments

All molecular copy numbers used in our tests have been generated by MATLAB [42] simulations of the discrete stochastic model dynamics using the Gillespie algorithm [4]. In general, a single realization of the process allows to mimic a single observation of the system giving rise to an experimental concentration data matrix 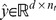. Here, 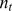 is the time discretization parameter and 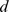 is the number of observable components of the state variable as defined by the state observation operator
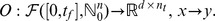



A 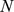-time repetition of the experimental observation of the system (or the computational realization of the stochastic process) then yields the sequence
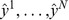
(25)


of data matrices. For each discrete time point 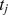 the data matrix 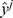 allows to calculate sample moments [62], [63]. For instance, the sample mean of the observables at time 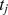 is given by
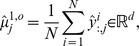



while the empirical covariance matrix of the observables at time 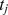 is given by
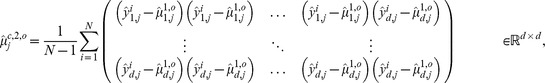



in which 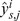, 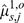 denote the 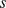-th components of the vectors 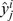, 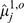. An alternative covariance matrix estimate that is more suitable if 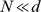 is not satisfied is given in [64]. In general, the data tensor 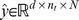 allows to compute the tupel
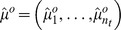



of length 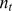, where 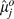 denotes the sample moments of the observable state components up to the order 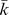 at time 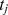. An example with 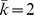 is
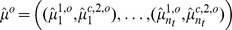



with sample mean vector 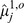 and sample covariance matrix 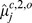 at time 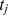.

### Cost Functions and Adjoint Method

If we split the state variable 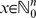 into the observable part 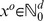 and the unobservable part 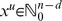 according to 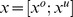, this separation carries over to the 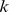-th order moments of 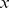 approximatively described by the ODE system (21), i.e., 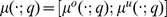. This is to be understood in the sense that there exists a (linear) splitting operator 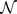 with
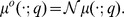



Then, in order to compare the parameter dependent solution component 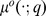 of the ODE system (21) with the available sample moment tuple 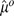 various distance measures
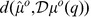
(26)


may be utilized where the time discretization operator 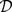 simply evaluates the time-dependent 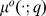 function at the discrete times 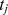 of (23), i.e.,
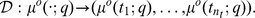



Examples for 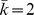 with 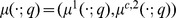 include
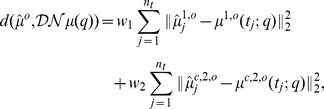


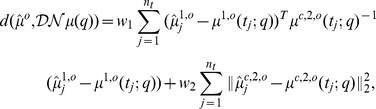


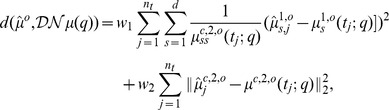



where, in general, 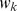 denotes the weight associated to the 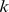-th order moment comparison. As shown in the results section the choice 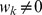 for 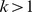 can make a decisive difference in the parameter inference problem for stochastic biological models. As lower order statistical moments in general are easier to approximate an ordering of the weights according to 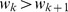 seems reasonable. In our tests we chose the weights by trial and error heuristics. Though we observed comparable performances for weights within a proper range, rules for the choice of 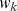 in dependence on 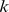 and the quality of data are desirable, see Discussion .

With respect to parameter inference the difference function (26) can be utilized in various manners. For instance, it can be minimized by deterministic or stochastic optimization routines, it can be incorporated as a cost function in approximate Bayesian methods or used in building Kalman filters or Luenberger type observers. In the context of gradient based optimization, the gradient information can be efficiently provided by means of the so-called adjoint technique whenever 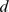 can be written as a parameter dependent 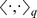 or parameter independent 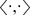 inner product of the residual 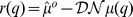 with itself, see Supporting Information S2.

## Supporting Information

Supporting Information S1 **Moment Equations for Test Models.**
(PDF)

Supporting Information S2 **Adjoint Method for Gradient based Optimization.**
(PDF)
