# Supplementary material for: Moment Fitting for Parameter Inference in Repeatedly and Partially Observed Stochastic Biological Models
Source: PLoS One. 2012 Aug 10;7(8):e43001. doi: 10.1371/journal.pone.0043001 (PMC3416831; doi:10.1371/journal.pone.0043001)
Supplement: Supporting Information S1 — Moment Equations for Test Models. (PDF) [file pone.0043001.s001.pdf]

S1 - Moment Equations for Test Models

Linear Birth Death Process

Based on the Kolmogorov differential equation

$$\frac{\partial \pi}{\partial t}(x,t) = q_1(x-1)\pi(x-1,t) - (q_1+q_2)x\pi(x,t) + q_2(x+1)\pi(x+1,t),$$

the time evolution of the mean

$$\mu^1(t) = E[x(t)] = \sum_{\tilde{x}\in\mathcal{X}} \tilde{x}\pi(\tilde{x},t)$$

is described by

$$\begin{aligned}
\frac{\partial}{\partial t}\mu^1(t) &= \sum_{\tilde{x}\in\mathcal{X}} \tilde{x}\frac{\partial \pi}{\partial t}(\tilde{x},t) \\
&= \sum_{\tilde{x}\in\mathcal{X}} \big\{q_1\tilde{x}(\tilde{x}-1)\pi(\tilde{x}-1,t) - (q_1+q_2)\tilde{x}^2\pi(\tilde{x},t) \\
&\quad +q_2\tilde{x}(\tilde{x}+1)\pi(\tilde{x}+1,t)\} \\
&= \sum_{\tilde{x}\in\mathcal{X}} \big\{q_1(\tilde{x}+1)\tilde{x}\pi(\tilde{x},t) - (q_1+q_2)\tilde{x}^2\pi(\tilde{x},t) \\
&\quad +q_2(\tilde{x}-1)\tilde{x}\pi(\tilde{x},t)\} \\
&= (q_1-q_2)\mu^1(t).
\end{aligned} \tag{1}$$

Furthermore, the time evolution of the second moment

$$\mu^2(t) = \sum_{\tilde{x}\in\mathcal{X}} \tilde{x}^2\pi(\tilde{x},t)$$

is given by

$$\begin{aligned}
\frac{\partial}{\partial t}\mu^2(t) &= \sum_{\tilde{x}\in\mathcal{X}} \tilde{x}^2\frac{\partial}{\partial t}\pi(\tilde{x},t) \\
&= \sum_{\tilde{x}\in\mathcal{X}} \big\{q_1\tilde{x}^2(\tilde{x}-1)\pi(\tilde{x}-1,t) - (q_1+q_2)\tilde{x}^3\pi(\tilde{x},t) \\
&\quad +q_2\tilde{x}^2(\tilde{x}+1)\pi(\tilde{x}+1,t)\big\} \\
&= \sum_{\tilde{x}\in\mathcal{X}} \big\{q_1(\tilde{x}+1)^2\tilde{x}\pi(\tilde{x},t) - (q_1+q_2)\tilde{x}^3\pi(\tilde{x},t) \\
&\quad +q_2(\tilde{x}-1)^2\tilde{x}\pi(\tilde{x},t)\big\} \\
&= 2(q_1-q_2)\mu^2(t) + (q_1+q_2)\mu^1(t).
\end{aligned} \tag{2}$$

Finally, the second central moment (the variance)

$$\mu^{c,2}(t) = \mu^2(t) - \mu^1(t)\cdot\mu^1(t)$$

satisfies

$$\begin{aligned}
\frac{\partial}{\partial t}\mu^{c,2}(t) &= \frac{\partial}{\partial t}\mu^2(t) - 2\mu^1(t)\frac{\partial}{\partial t}\mu^1(t) \\
&= 2(q_1-q_2)\mu^2(t) + (q_1+q_2)\mu^1(t) - 2\mu^1(t)(q_1-q_2)\mu^1(t) \\
&= 2(q_1-q_2)\mu^{c,2}(t) + (q_1+q_2)\mu^1(t).
\end{aligned} \tag{3}$$

If the diffusion approximation is chosen as modelling approach, the associated Fokker Planck equation reads as

$$\frac{\partial}{\partial t}p(\chi,t) = -\frac{\partial}{\partial \chi}\left\{(q_1-q_2)\chi p(\chi,t)\right\} + \frac{1}{2}\frac{\partial^2}{\partial \chi^2}\left\{(q_1+q_2)\chi p(\chi,t)\right\}.$$

Based on the Fokker Planck equation the time evolution of the first two moments

$$\int_{-\infty}^{\infty} \tilde{\chi}^k p(\tilde{\chi},t)\, d\tilde{\chi},\ k=1,2$$

is described by

$$\begin{aligned}
\frac{\partial}{\partial t}\mu^1[\chi(t)] &= E[(q_1-q_2)\chi(t)] = (q_1-q_2)\mu^1[\chi(t)],\\
\frac{\partial}{\partial t}\mu^2[\chi(t)] &= 2(q_1-q_2)\mu^2[\chi(t)] + (q_1+q_2)\mu^1[\chi(t)],
\end{aligned}$$

which is identical to (1), (2). Finally, also the linear noise approximation leads to the ODE system (1), (3) for the description of the time courses of μ^1 and $\mu^{c,2}$.

Dimerisation Process

The Fokker Planck equation of the diffusion modelling approach is given by

$$\frac{\partial}{\partial t}p(\chi,t) = -\frac{\partial}{\partial \chi}\left\{(-q_1\chi(\chi-1)+q_2(\chi_0-\chi))p(\chi,t)\right\} + \frac{1}{2}\frac{\partial^2}{\partial \chi^2}\left\{(2q_1\chi(\chi-1)+2q_2(\chi_0-\chi))p(\chi,t)\right\}$$

and implies

$$\begin{aligned}
\frac{\partial}{\partial t}\mu^1[\chi(t)] &= E[(-q_1\chi(\chi-1)+q_2(\chi_0-\chi))]\\
&= -q_1E[\chi^2(t)-\chi(t)] + q_2(\chi_0-E[\chi(t)])\\
&= q_1\left[\mu^1[\chi(t)]-\mu^2[\chi(t)]\right] + q_2(\chi_0-\mu^1[\chi(t)])\\
&= q_1\left[\mu^1[\chi(t)]-\mu^1[\chi(t)]\cdot\mu^1[\chi(t)]\right] + q_2(\chi_0-\mu^1[\chi(t)]) - q_1\mu^{c,2}[\chi(t)]
\end{aligned}$$

and

$$\begin{aligned}
\frac{\partial}{\partial t}\mu^2[\chi(t)] &= 2E[\chi(t)(-q_1\chi(t)(\chi(t)-1)+q_2(\chi_0-\chi(t)))] + E[2q_1\chi(t)(\chi(t)-1)+2q_2(\chi_0-\chi(t))]\\
&= 2E[-q_1\chi^3(t)+q_1\chi^2(t)+q_2\chi_0\chi(t)-q_2\chi^2(t)] + E[2q_1\chi^2(t)-2q_1\chi(t)+2q_2\chi_0-2q_2\chi(t)]\\
&= q_1\left[-2\mu^3[\chi(t)]+4\mu^2[\chi(t)]-2\mu^1[\chi(t)]\right] + q_2\left[2\chi_0(\mu^1[\chi(t)]+1)-2\mu^2[\chi(t)]-2\mu^1[\chi(t)]\right].
\end{aligned}$$

This is exactly the same set of moment ODEs if the derivation alternatively is based on the discrete state space and the Kolmogorov equation

$$\frac{\partial \pi}{\partial t}(x,t) = q_1\frac{(x+2)(x+1)}{2}\pi(x+2,t) - \left(q_1\frac{x(x-1)}{2} + q_2\frac{x_0-x}{2}\right)\pi(x,t) + q_2\frac{x_0-x+2}{2}\pi(x-2,t).$$

The corresponding equation for the second central moment (the variance) reads as

$$\begin{aligned}
\frac{\partial}{\partial t}\mu^{c,2}(t) &= \frac{\partial}{\partial t}\mu^2(t) - 2\mu^1(t)\frac{\partial}{\partial t}\mu^1(t)\\
&= q_1\left[-2\mu^3[\chi(t)]+4\mu^2[\chi(t)]-2\mu^1[\chi(t)]\right] + q_2\left[2\chi_0(\mu^1[\chi(t)]+1)-2\mu^2[\chi(t)]-2\mu^1[\chi(t)]\right]\\
&\quad +2q_1\left[\mu^2[\chi(t)]\cdot\mu^1[\chi(t)]-\mu^1[\chi(t)]\cdot\mu^1[\chi(t)]\right] - 2q_2(\chi_0-\mu^1[\chi(t)])\cdot\mu^1[\chi(t)]\\
&= 2q_1\left[-\mu^3[\chi(t)]+2\mu^{c,2}[\chi(t)]-\mu^1[\chi(t)]+\mu^1[\chi(t)]\cdot\mu^1[\chi(t)]+\mu^{c,2}[\chi(t)]\cdot\mu^1[\chi(t)]\right]\\
&\quad +2q_1\mu^1[\chi(t)]\cdot\mu^1[\chi(t)]\cdot\mu^1[\chi(t)] + 2q_2\left[\chi_0-\mu^1[\chi(t)]-\mu^{c,2}[\chi(t)]\right].
\end{aligned}$$

In order to eliminate the dependency of $\mu^1[\chi(t)]$, $\mu^{c,2}[\chi(t)]$ on $\mu^3[\chi(t)]$ one option is the normal closure approximation [1], [2], [3], [4], [5], which sets

$$\mu^3[\chi(t)] = 3\mu^{c,2}[\chi(t)] \cdot \mu^1[\chi(t)] + \mu^1[\chi(t)] \cdot \mu^1[\chi(t)] \cdot \mu^1[\chi(t)].$$

The resulting *approximative* ODE system

$$\mu_t(t) = F(\mu(t), q), \tag{4}$$

for $\mu(t; q) = [\mu^1[\chi(t)], \mu^{c,2}[\chi(t)]]^T$ is given

$$\begin{aligned}
\frac{\partial}{\partial t}\mu^1[\chi(t)] &= q_1\mu^1[\chi(t)]\left[1 - \mu^1[\chi(t)]\right] + q_2(\chi_0 - \mu^1[\chi(t)]) - q_1\mu^{c,2}[\chi(t)] \\
\frac{\partial}{\partial t}\mu^{c,2}[\chi(t)] &= -2q_1(2\mu^1[\chi(t)] + 2)\mu^{c,2}[\chi(t)] - 2q_2\mu^{c,2}[\chi(t)] \\
&\quad +2q_1\mu^1[\chi(t)](\mu^1[\chi(t)] - 1) + 2q_2(\chi_0 - \mu^1[\chi(t)]).
\end{aligned}$$

An alternative *approximative* ODE system (4) for $m(t) = [\mu^1(t), \mu^{c,2}(t)]^T$ can be derived from the linear noise approximation which reads as

$$\begin{aligned}
\frac{\partial}{\partial t}\mu^1[x(t)] &= q_1\mu^1[x(t)](1 - \mu^1[x(t)]) + q_2(x_0 - \mu^1[x(t)]), \\
\frac{\partial}{\partial t}\mu^{c,2}[x(t)] &= -2q_1(2\mu^1[x(t)] - 1)\mu^{c,2}[x(t)] + 2q_2(x_0 - 1)\mu^{c,2}[x(t)] \\
&\quad +2q_1\mu^1[x(t)](\mu^1[x(t)] - 1) + 2q_2(\chi_0 - \mu^1[x(t)]).
\end{aligned}$$

References

1. Lee CH, Kyeong-Hun Kim KH, Kim P (2009) A moment closure method for stochastic reaction networks. Journal of Chemical Physics 130: 813-819.

2. Engblom S (2006) Computing the moments of high dimensional solutions of the master equation. Applied Mathematics and Computation 180: 498-515.

3. Gillespie CS (2009) Moment-closure approximations for mass-action models. IET Systems Biology 3: 52-58.

4. Milner P, Gillespie CS, Wilkinson DJ (2011) Moment closure approximations for stochastic kinetic models with rational rate laws. Mathematical Biosciences 231: 99-104.

5. Matis TI, Guardiola IG (2010) Achieving moment closure through cumulant neglect. The Mathematica Journal 12.
