# Supplementary material for: Moment Fitting for Parameter Inference in Repeatedly and Partially Observed Stochastic Biological Models
Source: PLoS One. 2012 Aug 10;7(8):e43001. doi: 10.1371/journal.pone.0043001 (PMC3416831; doi:10.1371/journal.pone.0043001)
Supplement: Supporting Information S2 — Adjoint Method for Gradient based Optimization. (PDF) [file pone.0043001.s002.pdf]

S2 - Adjoint Method for Gradient based Optimization

For simplicity, consider a cost function d that can be written as a parameter independent $\langle\cdot,\cdot\rangle$ inner product of the residual $r(q) = \hat{\mu}^o - \mathcal{D}\mathcal{N}\mu(q)$ with itself. Then, the derivative of

$$d(\hat{\mu}^o, \mathcal{D}\mathcal{N}\mu(q)) = \langle r(q), r(q)\rangle = \langle \hat{\mu}^o - \mathcal{D}\mathcal{N}\mu(q), \hat{\mu}^o - \mathcal{D}\mathcal{N}\mu(q)\rangle$$

in direction of the j-th unit vector e^j of $\mathbb{R}^l$ in first place is given by

$$\frac{\partial}{\partial v} d(\hat{\mu}^o, \mathcal{D}\mathcal{N}\mu(q)) = -2\langle r(q), \mathcal{D}\mathcal{N}m^j(q)\rangle,$$

where $m^j(t;q)$ denotes the solution of the linear ODE system

$$\frac{\partial}{\partial t} m^j(t) = F_\mu(\mu(t), q)m^j(t) + F_q(\mu(t), q)e^j, \; m^j(0) = 0 \tag{1}$$

obtained from the moment ODE system

$$\mu_t(t) = F(\mu(t), q) \tag{2}$$

by linearization. Introducing the associated adjoint system

$$\frac{\partial}{\partial t} u(t) = -F_\mu(\mu(t), q)^T u(t) + \mathcal{N}^*\mathcal{D}^* r(q), \; u(t_f) = 0, \tag{3}$$

where F_μ^T denotes the transposed matrix of the Jacobian F_μ and $\mathcal{N}^*\mathcal{D}^*$ denotes the adoint operator of $\mathcal{D}\mathcal{N}$, the derivative can equivalently be expressed as

$$\frac{\partial}{\partial v} d(\hat{\mu}^o, \mathcal{D}\mathcal{N}\mu(q)) = 2\left(u, F_q(\mu(q), q)e^j\right). \tag{4}$$

This follows from

$$\begin{aligned}
\langle r(q), \mathcal{D}\mathcal{N}m^j(q)\rangle &= \left(\mathcal{N}^*\mathcal{D}^* r(q), m^j(q)\right) \\
&= \left(\frac{\partial}{\partial t} u(q) + F_\mu(\mu(q), q)^T u(q), m^j(q)\right) \\
&= \left(\frac{\partial}{\partial t} u(q), m^j(q)\right) + \left(u(q), F_\mu(\mu(q), q)m^j(q)\right) \\
&= -\left(u(q), \frac{\partial}{\partial t} m^j(q)\right) + \left(u(q), F_\mu(\mu(q), q)m^j(q)\right) \\
&= -\left(u(q), F_q(\mu(q), q)e^j\right)
\end{aligned}$$

where $(\cdot,\cdot)$ denotes the innner product in $L^2(0, t_f)$. Building the gradient information via (1) requires to solve the linearized system l times, i.e., one time for each direction e^j. The computational advantage of the adjoint approach is that only the j-independent linearized system (3) has to be solved (backwards in time) in order to then build (4) for $j = 1, \ldots, l$.

Given function values $f = (f_0, \ldots, f_{n_t})^T$ at discrete time points, the adjoint of $\mathcal{D}$ is defined by

$$\mathcal{D}^* f = \sum_{j=1}^{n_t} f_j \delta(t - t_j),$$

where the δ-function satisfies $\delta(0) = 1$ and $\delta(\tau) = 0$ for $\tau \neq 0$. The adjoint of the embedding operator $\mathcal{N}$ maps moment expressions for observables to moment expressions of the full state space by introducing zeros whenever an unobservable is involved. For instance, with $\bar{k} = 1$ we have

$$\mathcal{N}^* : \mathcal{F}([0, t_f], \mathbb{R}^d) \to \mathcal{F}([0, t_f], \mathbb{R}^n), \mu^{1,o}(\cdot; q) \to [\mu^{1,o}(\cdot; q); 0].$$

The key ingredient to (3) is the Jacobian matrix $F_\mu(\mu(t; q), q)$ which can be optained manually or by symbolic computation tools to be evaluated at the solution $\mu(t; q)$ of (2) for the current parameter guess q. The system (3) can be solved by standard ODE solvers after transformation to the time variable $\tau = t_f - t$ in order to obtain a (well-posed) system forward in time.
